# Association between clinical and environmental factors and the gut microbiota profiles in young South African children

**DOI:** 10.1038/s41598-021-95409-5

**Published:** 2021-08-05

**Authors:** Kristien Nel Van Zyl, Andrew C. Whitelaw, Anneke C. Hesseling, James A. Seddon, Anne-Marie Demers, Mae Newton-Foot

**Affiliations:** 1 Division of Medical Microbiology, Department of Pathology, Stellenbosch University, Stellenbosch, South Africa; 2 National Health Laboratory Service, Tygerberg Hospital, Cape Town, South Africa; 3 African Microbiome Institute, Stellenbosch University, Stellenbosch, South Africa; 4 Desmond Tutu TB Centre, Department of Paediatrics and Child Health, Stellenbosch University, Stellenbosch, South Africa; 5 Department of Infectious Diseases, Imperial College London, London, UK

**Keywords:** Microbial communities, Paediatric research

## Abstract

Differences in the microbiota in populations over age and geographical locations complicate cross-study comparisons, and it is therefore essential to describe the baseline or control microbiota in each population. This includes the determination of the influence of demographic, clinical and environmental factors on the microbiota in a setting, and elucidates possible bias introduced by these factors, prior to further investigations. Little is known about the microbiota of children in South Africa after infancy. We provide a detailed description of the gut microbiota profiles of children from urban Cape Town and describe the influences of various clinical and environmental factors in different age groups during the first 5 years of life. *Prevotella* was the most common genus identified in the participants, and after infancy, the gut bacteria were dominated by Firmicutes and Bacteroidetes. In this setting, children exposed to antibiotics and indoor cooking fires were at the most risk for dysbiosis, showing significant losses in gut bacterial diversity.

## Introduction

There is a growing appreciation of the influence of the human microbiome on health. The gut microbiome, in particular, has been linked to many functions that are important for good health, such as aiding the development of a healthy immune system, protecting the gut against pathogen-adherence and producing antimicrobial compounds^1–3^.

Disruption or dysbiosis of the gut microbial community (microbiota) has a significant impact on human health and has been linked to multiple metabolic, immunological, and developmental disorders^4^. This is especially significant in children, where the first 1000 days of life from conception to the age of two are crucial for the development of various developmental pathways, including immunity and metabolism^5,6^. Dysbiosis in early life can disrupt these pathways and possibly have long term implications for growth and health^7^. For example, antibiotic use has been associated with increased risk of asthma, allergies, eczema, necrotizing enterocolitis (NEC) and inflammatory bowel disease (IBD)^8^. Infants delivered via caesarean section have an increased risk for atopic diseases such as asthma, type 1 diabetes, coeliac disease and food allergies compared to those delivered vaginally^8,9^. Conversely, probiotic exposure and breastfeeding have been associated with decreased incidence of asthma, food-related allergies and IBD^8^. While it is not always possible to identify the source of dysbiosis, associations between specific microorganisms and dysbiosis have been found in several diseases in children, including NEC, IBD, obesity, atopy and asthma, and autism-spectrum disorder^10^.

Knowing which factors may influence the microbiota could inform dysbiosis-prevention strategies. However, microbiota profiles vary with age and health status and are heterogeneous across geographical regions. It is therefore important to describe the baseline microbiota in different populations and to investigate the influence of setting-specific clinical, environmental, socio-economic and other factors on the microbiota.

There is a marked difference between the microbiota in urban westernised populations, and those in rural and developing settings, in adults and children^11–14^. In South Africa, the microbiota of adults represents a transitional state between the typical profiles in western populations and other African populations^15^. There is little known about the microbiota of children in South Africa, especially after infancy. A small number of South African studies have recently explored the human gut microbiota in young children, but they have either been birth cohorts evaluating infants under the age of 1 year^16,17^ or have included only children with specific diseases or disorders^18,19^.

The aim of this study was to characterize the bacterial gut microbiota profiles of children below 5 years of age living in urban Cape Town communities. We investigated the influence of various clinical and environmental factors on the gut bacterial profiles of these children and sought to describe differences in bacterial microbiota in different age groups during the first 5 years of life.

## Materials and methods

### Study design

This study was nested in the ongoing phase III, cluster randomised, double-blinded, placebo-controlled tuberculosis child multidrug-resistant preventive therapy (TB-CHAMP) trial (Registration date: 31/03/2016; Registration number: ISRCTN92634082; http://www.isrctn.com/ISRCTN92634082) which aims to assess the efficacy and safety of levofloxacin as preventive therapy in children exposed to multidrug-resistant (MDR) tuberculosis (TB) within the household.

Participants were recruited from urban communities (often living in over-crowded and/or informal housing conditions) in the Cape Town metropolitan area (Western Cape, South Africa) which has a population of 4 million people. The TB incidence rate in the Western Cape was 681/100,000 in 2015^20^. The antenatal HIV prevalence was 15.9% for the province in 2017^21^. In the same year, the South African Medical Research Council estimated infant mortality rate in South Africa at 23 per 1000 live births, whereas the under-five mortality rate was 32 per 1000 births^22^.

Participants were eligible if they were aged < 5 years and were a household contact of an enrolled adult index case (≥ 18 years of age) with bacteriologically confirmed pulmonary MDR-TB and if informed consent from the parent or legal guardian of the child was obtained for HIV testing and enrolment in the study. Exclusion criteria included presenting with TB disease at enrolment, receiving isoniazid or any fluoroquinolone treatment for ≥ 14 days at screening, receiving TB treatment in the previous 12 months, known exposure to an isoniazid-susceptible TB index case and those diagnosed with Myasthenia gravis or Guillain–Barré syndrome.

Stool samples were collected from child participants at their baseline trial visit (before randomisation into the treatment or placebo groups), from November 2017 until July 2019, and subjected to 16S rRNA gene amplicon sequencing to characterise the bacterial microbiota. Approval to conduct the TB-CHAMP trial was obtained from the Stellenbosch University Human Research Ethics Committee (SU-HREC, M16/02/009), South African Health Products Regulatory Authority (20160128) and local health authorities. This sub-study was conducted at Stellenbosch University in Cape Town only and ethical approval was obtained from the SU-HREC (S18/02/031). The research from both the parent trial and this sub-study were conducted as outlined by Stellenbosch University’s Policy for responsible research conduct in human participants and guidelines set out by the World Health Organization (WHO) and the Declaration of Helsinki.

### Demographic, clinical and environmental data collection

Extensive systematic data collection was undertaken at the baseline visit through clinical interview, questionnaire to the household, and physical examination. Participant demographics and clinical and environmental factors such as HIV status, medical history (including mode of birth, antibiotic use, deworming and hospitalisation), exposure to cigarette smoke, indoor cooking fires, breastfeeding and exposure to pets and day-care was recorded. Mid-upper arm circumference (MUAC) scores were used as an indicator of malnutrition in participants > 6 months. A MUAC of > 13.5 cm indicates a low risk for malnutrition, while a MUAC between 12.5 and 13.5 cm indicates mild risk for malnutrition. The following were used as indicators of socio-economic status: housing structure type, ablution type and drinking water supply. Where relevant, children were grouped into three age bands: A (0–1 years), B (> 1 to 2 years) and C (> 2 to < 5 years), to account for the highly evolving nature of the gut microbiota during early childhood.

### Sample collection

Stool samples (1 per child) were collected in sterile 25 ml faecal containers with spoons (Lasec, South Africa) without preservative and transported to the laboratory in cooler boxes with ice packs within 72 h, where they were homogenised and immediately stored at − 80 °C. Samples were stored on ice packs (n = 49) or in a fridge at 2–8 °C (n = 59) before transport. In a small number of cases (n = 8) no such facilities were available, and samples were stored at room temperature. Sample weight and consistency prior to homogenisation were also recorded.

### DNA extraction and sequencing

DNA was extracted using the QIAamp PowerFecal DNA Isolation Kit (Qiagen, Germany) according to the manufacturer’s instructions. Samples that did not meet the purity requirements of the sequencing provider were subjected to ethanol-based purification. The extracted DNA was frozen at − 20 °C until delivery on ice to the Centre for Proteomic and Genomic Research (CPGR) where 16S rRNA gene amplicon sequencing was performed on the Illumina MiSeq platform. Previously published primers^16^ were used to target the V4 hypervariable region. The MiSeq Reagent v3 Kit (600 cycles) was used to generate sequencing libraries, which were spiked with 10% of a 5 pM PhiX sequencing control. The ZymoBIOMICS Microbial Community DNA standard (Zymo Research, USA) and batched negative extraction controls were included in the sequencing run, which was set to produce 2 × 200 bp paired-end reads. The DNA purity requirements and sequencing controls are described in detail in the “Supplementary material”.

### Sequence analysis and statistical testing

#### Sequence quality analysis

De-multiplexed paired-end FASTQ sequences were imported to and analysed with the Quantitative Insights Into Microbial Ecology (QIIME2 2020.8) bioinformatics platform^23^ on the Stellenbosch University high performance computing cluster (HPC) 2 (http://www.sun.ac.za/hpc). Primer sequences were removed with cutadapt^24^, followed by error correction, quality filtering and chimera removal using the dada2 plug-in^25^.

#### Taxonomic profiling

A Naïve Bayes classifier trained on the SILVA 138 99% OTU V4 region database (https://www.arb-silva.de/silva-license-information/) was used to assign taxonomy^26,27^. Taxonomic features appearing in less than five samples, as well as unassigned and non-bacterial features were filtered prior to visualizing the taxonomy bar plots.

#### Alpha and beta diversity testing

Rarefaction plots were generated with feature frequency and Shannon diversity, and five samples were excluded from all alpha and beta diversity analyses after rarefying to a sequencing depth of 52,653. Univariate analysis was performed for individual factors, samples with no data were automatically excluded and the p value for significance was set at 0.05 for all calculations. The evenness and richness (alpha diversity) within groups (based on the factors defined under the demographic, clinical and environmental data section) was calculated using the Shannon (H) and Faith’s Phylogenetic Diversity (PD) diversity metrics^28,29^ and statistical significance between groups was determined using Kruskal–Wallis pairwise tests and Benjamini–Hochberg False Discovery Rate (BH-FDR) multiple test correction where appropriate^30,31^. Spearman correlation analysis was performed for numerical data^32^. The differences in microbial communities between groups were investigated by principal coordinate analysis (PCoA) using the Bray–Curtis, and unweighted and weighted UniFrac dissimilarity metrics^33,34^. Significance of differences were determined by PERMANOVA^35^ with BH-FDR adjustment where appropriate.

#### Differential abundance analysis

Analysis of composition of microbiomes (ANCOM)^36^ was used to identify differentially abundant features at genus level for factors such as age, medication use, method of birth, breastfeeding and exposure to pets and smoke. The selection of factors to investigate for possible bacterial biomarkers was guided by clustering in the PCoA space following beta diversity analysis. For all ANCOM analyses, recommended filtering was performed to include only features present ≥ 20 times overall and in at least 25% of the samples, to remove noise caused by less abundant features.

## Results

### Participant demographics

Stool samples were collected from 116 of 218 children enrolled in Cape Town over the study period. Of these, after DNA extraction, only a single sample failed to meet the DNA purity requirements; the sample and corresponding participant data were excluded from analysis. The participant demographics and exposures to different clinical and environmental factors have been summarised in Tables 1 and 2 respectively, and additional details regarding breastfeeding habits and day-care exposure can be found in Supplementary Table S1. The median age of the children was 32 months (interquartile range [IQR]: 15–43). There were 60 (52.2%) boys, and only one confirmed HIV-positive child. Only four children over the age of 6 months had a MUAC of < 13.5 cm.

**Table 1:** Participant demographics and health status.

Demographics	
**Age**	Median 32 months (IQR: 15–43)
**Sex**	
Male	60 (52.2%)
Female	55 (47.8%)
**Method of birth**	
Normal vaginal delivery	80 (69.6%)
Caesarean section	32 (27.8%)
Unknown	3 (2.6%)
**Premature birth (< 37 weeks gestation)**	
Yes	17 (14.8%)
No	94 (81.7%)
Unknown	4 (3.5%)
**HIV status**	
Positive	1 (0.9%)
Negative	113 (98.2%)
Unknown	1 (0.9%)
**Maternal HIV status**	
Positive	44 (38.26%)
Negative	62 (53.91%)
Unknown	9 (7.83%)
**MUAC (children > 6 months)**	
> 13.5 cm	104 (96.3%)
≤ 13.5 cm	4 (3.7%)

**Table 2:** Participant exposure to clinical and environmental factors.

Clinical factors	
**Received antibiotics (< 2 weeks from baseline)**	
Beta-lactam (amoxicillin)	12 (10.4%)
Fluoroquinolone alone	3 (2.6%)
Fluoroquinolone + Cotrimoxazole + Ethambutol	1 (0.9%)
**Received antibiotics (< 6 months from baseline)**	
Yes	27 (23.5%)
No	72 (62.6%)
Unknown	16 (13.9%)
**Hospital admission (< 6 months from baseline)**	
Yes	7 (6.1%)
No	108 (93.9%)
**Hospital admission (first 6 months of life)**	
Yes	15 (13.1%)
No	98 (85.2%)
Unknown	2 (1.7%)
**Visit to traditional healer (< 3 years from baseline)**	
Yes	9 (7.8%)
No	106 (92.2%)
Dewormed* (< 6 months from baseline)	
Yes	26 (44.1%)
No	33 (55.9%)
**Vitamin A supplementation (< 6 months from baseline, in children > 6 months)**	
Yes	46 (42.6%)
No	51 (47.2%)
Unknown	11 (10.2%)

Environmental factors	
**Household cigarette smoke exposure**	
Yes	62 (53.9%)
No	50 (43.5%)
Unknown	3 (2.6%)
**Household indoor cooking fire exposure**	
Yes	38 (33%)
No	73 (63.5%)
Unknown	4 (3.5%)
**Pets (cats/dogs) in household**	
Yes	33 (28.7%)
No	78 (67.8%)
Unknown	4 (3.5%)

Socio-economic factors	
**Household structure**	
Tin shack	30 (26.1%)
Brick structure	75 (65.2%)
Wendy house	6 (5.2%)
Backyard shack of another house	1 (0.9%)
Prefabricated house	3 (2.6%)
**Ablution type**	
Bucket system	8 (7%)
Flush toilet in the house	66 (57.4%)
Shared flush toilet	18 (15.6%)
Flush toilet outside the house but for exclusive use	15 (13%)
Pit latrine	4 (3.5%)
Ventilated improved pit (VIP) latrine	4 (3.5%)
**Drinking water supply**	
Piped water from a public shared tap	23 (20%)
Piped water in the residence	84 (73%)
Piped water outside, but for exclusive use of the household	8 (7%)

Data expressed as number (%).

*Deworming was recorded on more than one case report form; data presented includes only participants for whom answers matched on both forms (n = 59).

### Sequencing and taxonomy profiles

**Figure 1:**
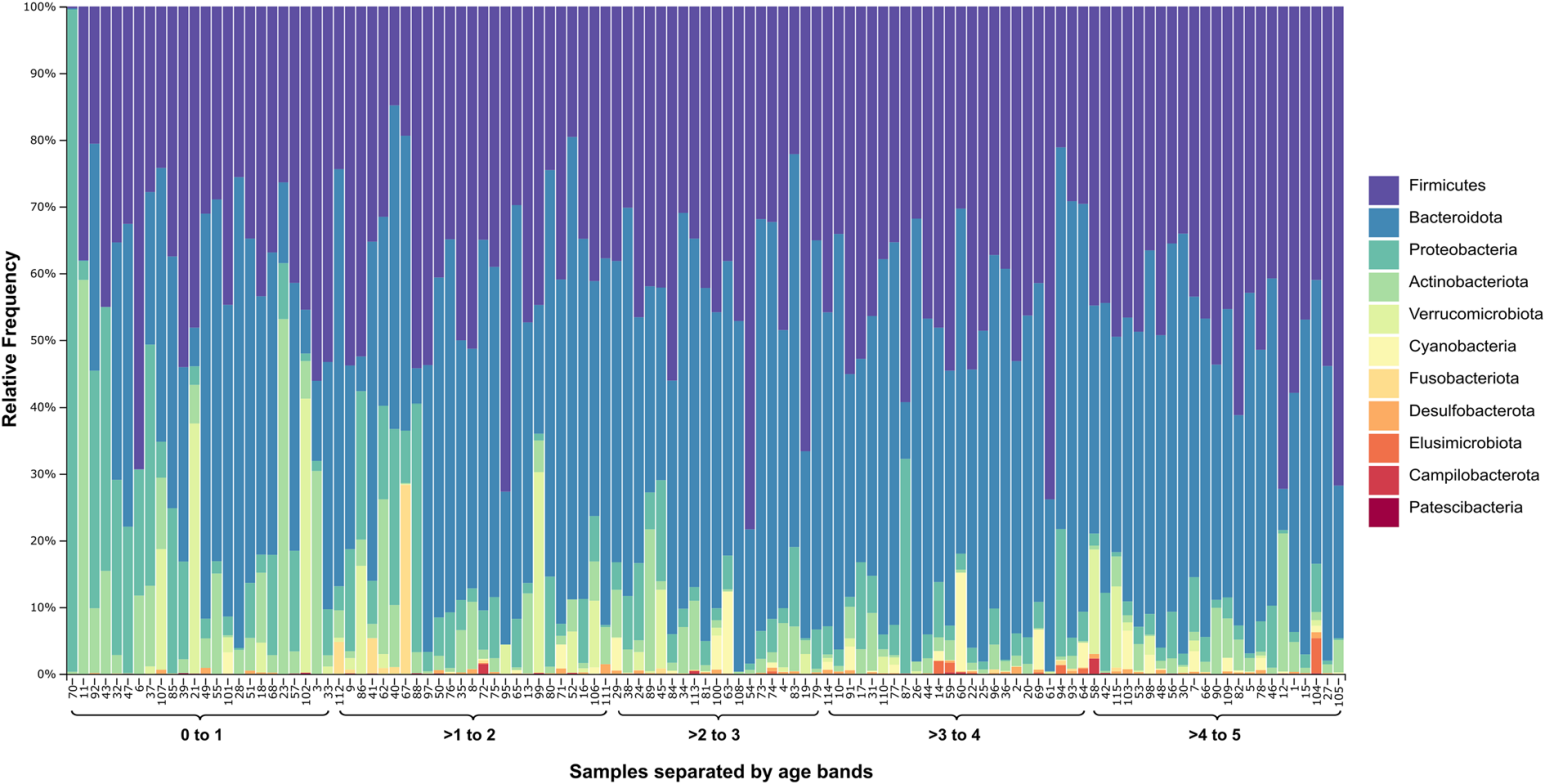
Taxonomic profiles of participant samples at phylum level. The brackets on the x-axis show the different age bands by year and samples within each band are ordered by increasing age. Features appearing in less than five samples, as well as unassigned and non-bacterial features were removed to simplify visualization.

**Figure 2:**
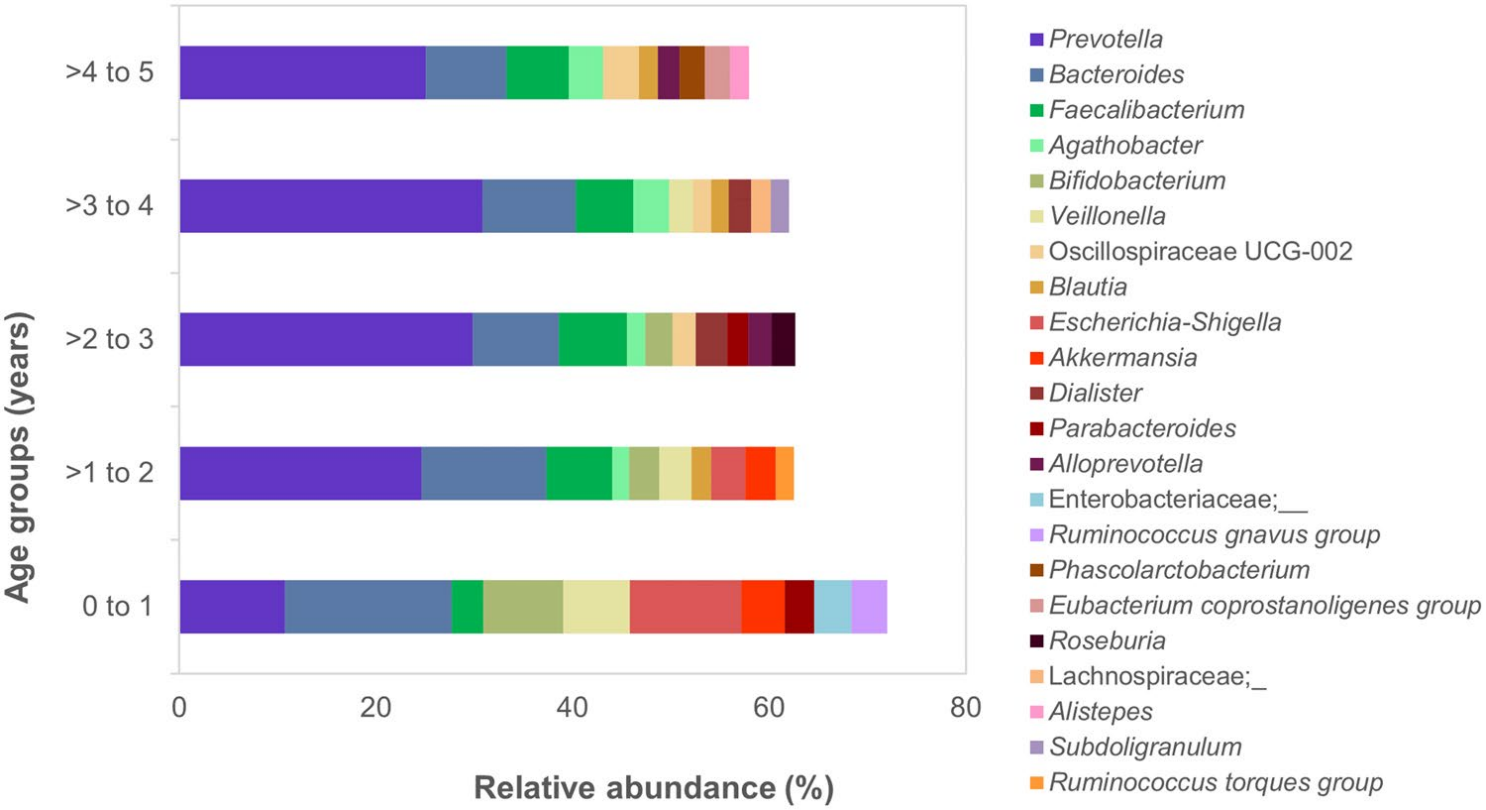
The relative abundance of the top ten genera in each of the five age groups.

A total of 9,481,364 paired reads were imported into QIIME2 and no trimming was performed due to high quality across all bases in both forward and reverse reads (median ≥ Q30). After quality filtering and chimera removal, a total of 8,019,511 paired reads remained (7,766,477 excluding controls, median: 67,517 reads/sample, IQR: 61,452–72,667). Taxonomic profiles were generated for each sample at phylum level and ordered by age group (Fig. 1). At phylum level, children younger than 1 year had a higher relative abundance of Proteobacteria (16.6%) and Actinobacteriota (9.8%) compared to those older than 2 years (Proteobacteria: 4.6%, Actinobacteriota: 2.9%). In children between the age of 2−5 years, the profiles were dominated by Firmicutes (45.1%) and Bacteroidota (44.9%). At genus level, there was a notable abundance of *Bifidobacterium*, *Escherichia*-*Shigella* and *Veillonella* in infants, whereas the most prevalent genus overall was *Prevotella* (Fig. 2).

### Alpha diversity

**Figure 3:**
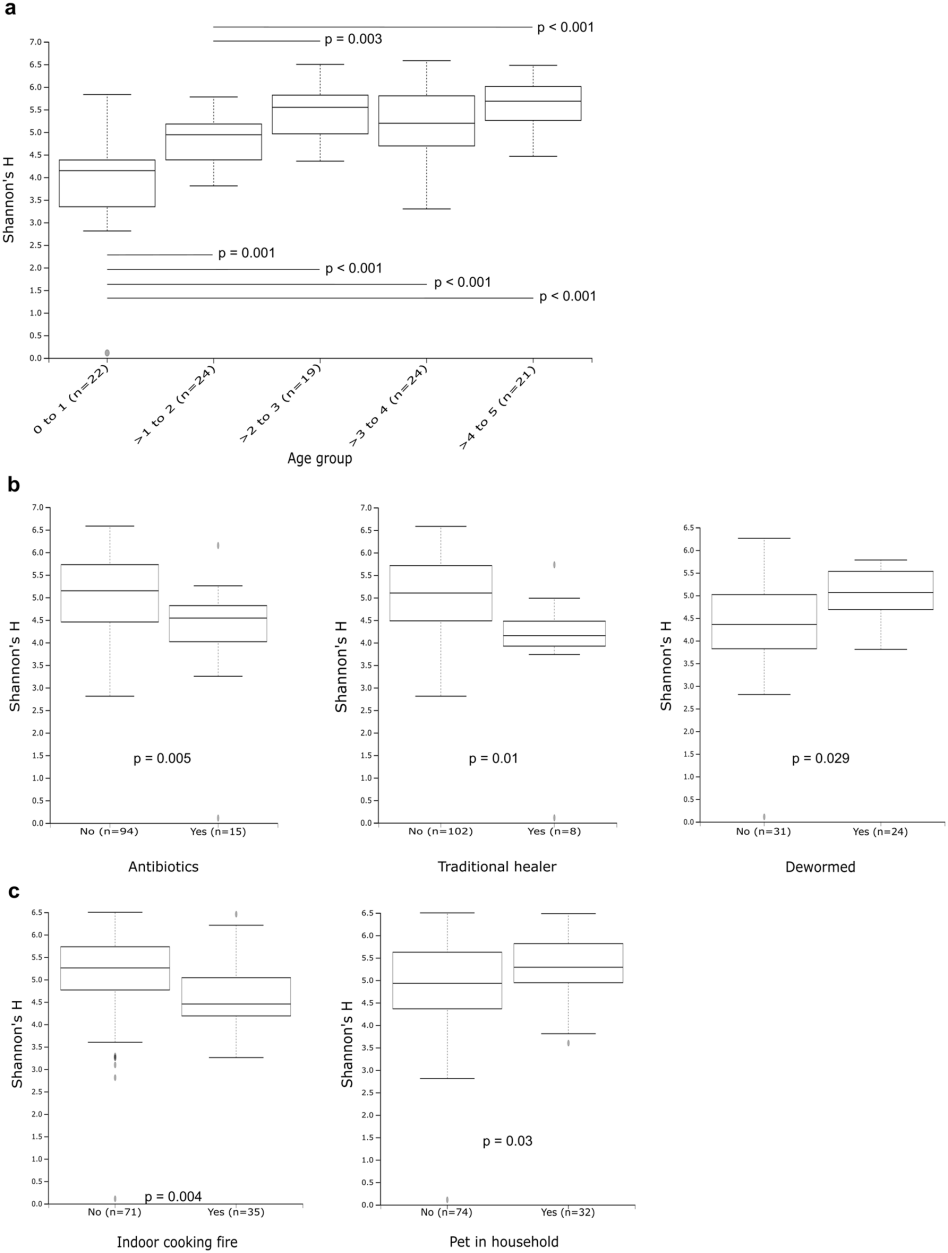
Shannon’s H alpha diversity plotted for significant factors (p < 0.05). (**a**) Age bands separated by year of life. Lines indicate significant differences between the groups at each end of the line. There were no significant differences between the groups representing the oldest three age bands. (**b**) Clinical factors, including receipt of antibiotics within 2 weeks from sample collection, treatment from traditional healer within 3 years from sample collection, and deworming within 6 months from sample collection. (**c**) Environmental factors, including exposure to an indoor cooking fire using paraffin or wood, and exposure to pets in the household (cats/dogs). Faith’s PD results have been summarised in Supplementary Table S2.

The influence of various factors on the richness and evenness of bacteria in samples was determined by calculating Shannon’s H and Faith’s PD indexes (Fig. 3, Supplementary Tables S2 and S3). An increase in richness and evenness was observed during the first 3 years of life, as shown by Shannon’s H index (Fig. 3A) and Faith’s PD (Supplementary Table S2). The upper three 1-year age bands did not differ significantly from each other, indicating a stabilization of within-sample diversity. There were no significant differences in alpha diversity due to sex (M/F) or due to the mother’s HIV status. The method of sample storage, sample consistency and sample weight did not impact alpha diversity.

For factors more directly linked to age, such as method of birth (caesarean (C/S) vs normal vaginal delivery (NVD)), feeding pattern and day-care exposure, the data were stratified into three age groups: group A (0–1; n = 24), group B (> 1 to 2; n = 25) and group C (> 2 to 5; n = 66). None of these age-related factors were associated with changes in alpha diversity in any of the age groups, including method of birth (C/S vs NVD), premature birth, breastfeeding in the first 6 months (including exclusive breastfeeding), duration of breastfeeding (in months) and age of solid food introduction. In addition, none of the factors relating to day-care exposure, including the age at which day-care was started, size of day-care groups or hours spent in day-care, were associated with differences in alpha diversity in the oldest age group (Supplementary Tables S2 and S3).

Alpha diversity was significantly lower in those who had received antibiotics in the 2 weeks prior to sample collection (H: Fig. 3B, p = 0.005), but no difference was seen between children who had or had not received antibiotics in the last 6 months. Children who had received treatment from a traditional healer in the last 3 years had lower diversity (Fig. 3B; p = 0.01), while those who had been dewormed in the 6 months prior to sample collection had significantly higher diversity (Fig. 3B; p = 0.029). Vitamin A supplementation in those older than 6 months, hospitalisation in the 6 months prior to sample collection and hospitalisation in the first 6 months of life did not affect alpha diversity.

While there were no significant differences between children exposed to cigarette smoke during pregnancy or in the household, those exposed to an indoor cooking fire using wood or paraffin (kerosene) had significantly lower diversity (Fig. 3C; p = 0.004). Children who lived in a home with pets (cats or dogs) had significantly higher diversity (Fig. 3C; p = 0.03). The type of housing structure, ablutions and drinking water supply did not affect alpha diversity.

### Beta diversity

**Figure 4:**
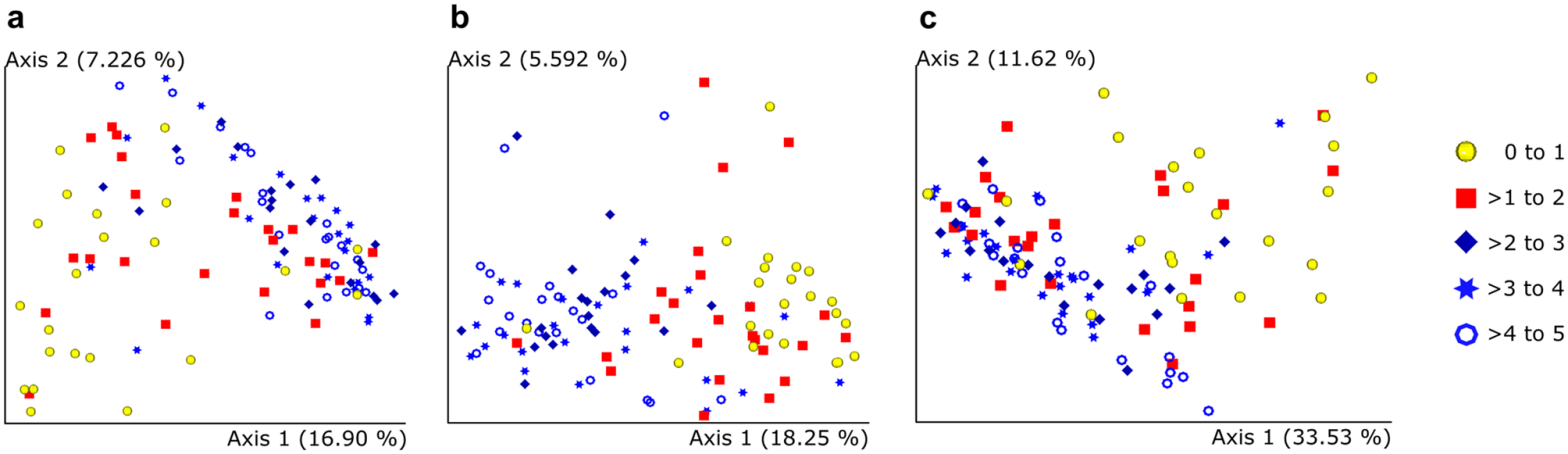
Two-dimensional PCoA plots based on three dissimilarity metrics. The three age groups are shown by colour. Yellow = A (0–1); Red = B (> 1 to 2); Blue = C (> 2 to 5). Group C is further subcategorized by shape. (**a**) Bray–Curtis, (**b**) Unweighted UniFrac, (**c**) Weighted UniFrac.

Like richness and evenness, differences in age were also associated with dissimilarity in the gut microbial community. The PCoA plots generated by the Bray–Curtis (BC), unweighted UniFrac (UWU) and weighted UniFrac (WU) dissimilarity metrics showed distinct clustering of samples in age groups A (0–1 years) and C (> 2 to 5 years), and a transitional cluster representing age group B (> 1 to 2 years) (Fig. 4). PERMANOVA testing showed that groups A and B were dissimilar to group C and each of its subgroups (> 2 to 3 years, > 3 to 4 years, and > 4 to 5 years), and that the subgroups of group C did not differ from each other.

There were significant differences in the bacterial communities when considering receipt of antibiotics in the 2 weeks prior to sample collection (BC: p = 0.001; UWU: p = 0.001; WU: p = 0.001), those who had visited a traditional healer in the last 3 years (BC: p = 0.009; UWU: p = 0.002; WU: p = 0.003), and deworming within 6 months (BC: p = 0.01; UWU: p = 0.011; WU: p = 0.039). Vitamin A supplementation trended toward, but did not significantly influence community dissimilarity (BC: p = 0.075; UWU: p = 0.067; WU: p = 0.286). Exposure to indoor cooking fires (BC: p = 0.036; UWU: p = 0.012; WU: p = 0.177), rather than cigarette smoke exposure (BC: p = 0.174; UWU: p = 0.366; WU: p = 0.133) was associated with a community shift, when considering most dissimilarity metrics. No other clinical, environmental or socio-economic factors influenced the gut microbial communities significantly. When stratified by age, no significant associations with any age-linked factors were identified.

### Differential abundance with ANCOM

**Figure 5:**
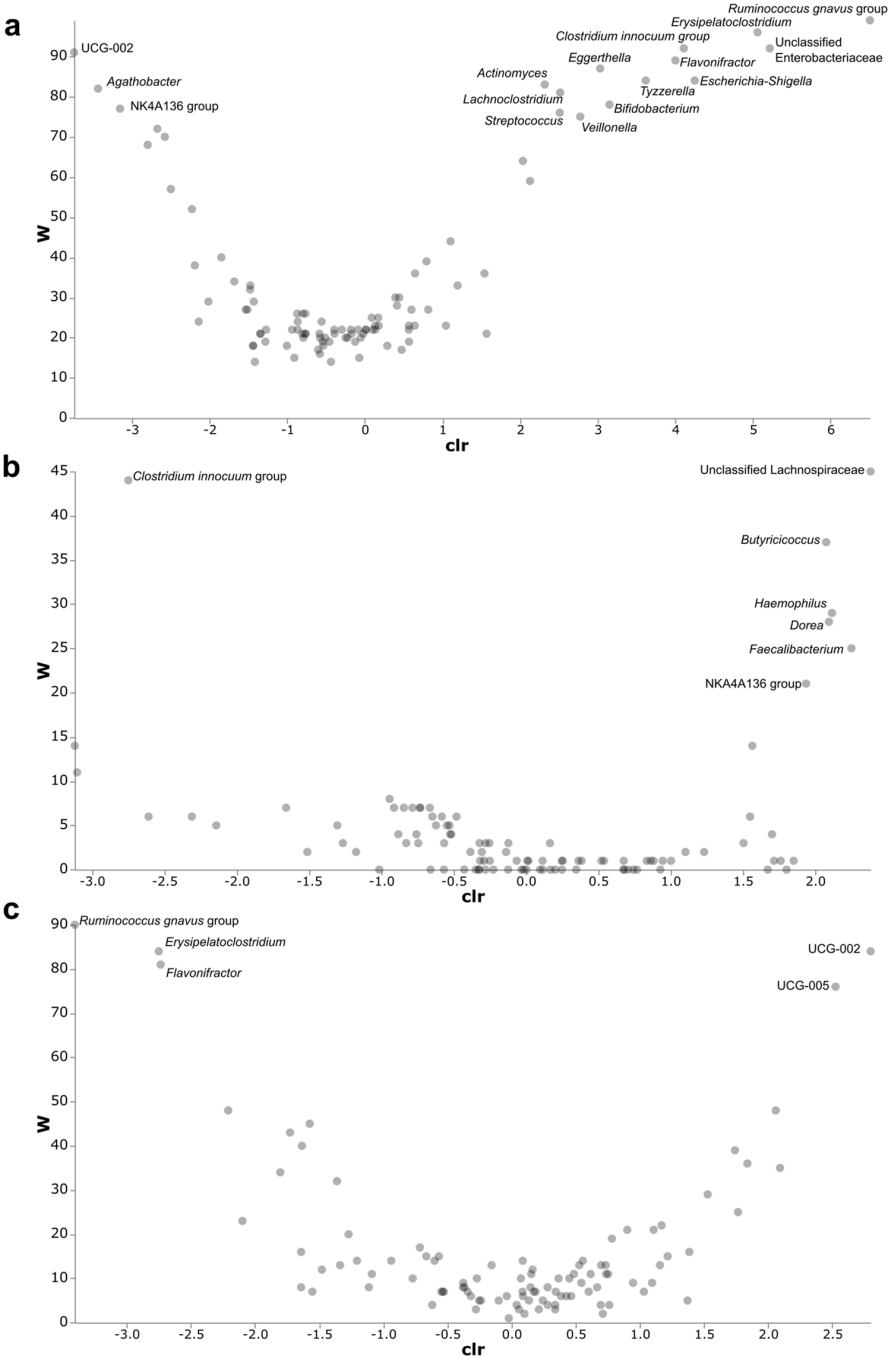
ANCOM generated volcano plots showing differentially abundant features between the age groups. W is the ANCOM test statistic and indicates the number of times the null hypothesis is rejected by the analysis. The higher W, the more likely a feature differs statistically. clr indicates the effect size change between the compared groups. Statistically significant features as identified by ANCOM have been labelled. (**a**) Comparison between groups A & C. A positive clr indicates higher abundance in A compared to C. (**b**) Comparison between groups A & B. A positive clr indicates higher abundance in B compared to A. (**c**) Comparison between groups B & C. A positive clr indicates higher abundance in C compared to B.

Differential abundance analysis was performed to identify possible bacterial biomarkers associated with the differences between groups following alpha- and beta-diversity analysis. These analyses support the observed age-related differences in alpha and beta diversity, and the transitional nature of the microbiome during infancy. Sixteen differentially abundant genera were identified between groups A and C, 7 between groups A and B, and 5 between groups B and C (Fig. 5). When comparing the age groups A and C, only three features (an uncultured genus-level group (UCG) from the Oscillospiraceae (UCG-002), *Agathobacter* and a genus group in the Lachnospiraceae family) were more abundant in the older age group. A number of features from the Actinobacteriota and Proteobacteria were significantly more abundant in the youngest age group. There was a significant increase in genera from the order Clostridia in group B, as compared to group A. The differentially abundant genera between groups B and C belong to the same phylum and therefore do not represent major global changes in the microbiota.

*Prevotella* was enriched in those not exposed to antibiotics in the 2 weeks prior to sample collection and *Haemophilus* was more abundant in those who had been dewormed. The differences in microbiota composition due to antibiotic treatment and deworming are shown in Supplementary Fig. S1. Other clinical and environmental factors that were associated with changes in diversity did not have any significant bacterial biomarkers. Factors that have been known to influence the microbiome in the first year of life, such as method of birth, prematurity and breastfeeding, were not associated with any differentially abundant features in group A or the older age groups.

## Discussion

This study characterized the bacterial gut microbiota profiles of children from urban communities in Cape Town, South Africa. Diversity and differential abundance analyses were performed in the context of various demographic, clinical and environmental factors. It was found that the richness and evenness of the bacterial microbiota in this population stabilize after infancy and that age is the main driver of differences between participants.

The gut microbiota of children in this study shows similar maturation patterns to those described previously in both urban and rural settings, where a shift occurs from Proteobacteria and Actinobacteria such as *Escherichia* and *Bifidobacterium* in early life, to Firmicutes/Bacteroidota dominated profiles represented by *Prevotella* and *Bacteroides* after infancy^11,37,38^. *Prevotella* was the most common genus in older children (> 2 years) in our study; this is comparable to studies done in other developing countries^11,39,40^. A recent South African study that evaluated the association between diet and atopic dermatitis in children found that the higher abundance of *Prevotella* in healthy controls may indicate a protective effect against atopic dermatitis^18^. There was no depletion of *Bacteroides*, as has been noted in some child and adult studies in rural African communities^41,42^. At phylum level, the microbiota profiles in this study were more similar to those of children in studies from the United States of America (USA) than those from Asia, Europe and Africa, with an equal distribution of Firmicutes and Bacteroidota^11,39,40^. This intermediate state between the microbial profiles of traditionally non-western microbiomes and western microbiomes has also been described in South African adults and has been attributed in part to urbanization and changes in diet^15^. A limitation of our study was that the dietary intake of the children was not recorded, and the impact of different diets on the gut microbiota could therefore not be investigated in this population. We found no significant differences in diversity or bacterial features when considering age-related factors known to influence the gut microbiota in early life, such as method of birth, prematurity, breastfeeding and day-care exposure. This may be related to the smaller number of participants when stratifying by age group, which limited the statistical analysis.

A significant increase in potential short-chain fatty acid (SCFA) producing taxa was noted after the first year of life, including members of the Lachnospiraceae, Oscillospiraceae and Ruminococcacaea families. *Agathobacter*, *Butyricicoccus* and Lachnospiraceae NK4A136 group were among the taxa which increased in abundance with age; these taxa have previously been identified as potential probiotic targets^43–45^. *Faecalibacterium*, another taxon associated with good health in adults^46^, represented a substantial proportion of the gut bacteria in this study.

Reduced microbial diversity was associated with antibiotic treatment within 2 weeks of sample collection, visiting traditional healers within 3 years and living in a home with an indoor wood or paraffin cooking fire, which may have placed children at risk for dysbiosis. It should be noted that all but one child in the small group who had visited traditional healers were below the age of two, and lower diversity in this univariate evaluation is thus likely to be driven by age. Of note, all of these children had visited the healers due to minor issues such as colic. In this study, only *Prevotella* was found to be significantly reduced in those who had recently taken antibiotics. No significant differences in microbial profiles or diversity were observed in children exposed to antibiotics within 6 months of sample collection, suggesting resilience and recovery of the microbiome after short-term antibiotic use. However, these children are exposed to MDR-TB in the household, may be biased toward living in poorer socio-economic conditions and they are likely to have a higher rate of antibiotic consumption and hospitalisation in the longer term, which may impact the microbiome. The possible selection of resistant organisms has not been considered here, but there is contradictory evidence regarding the long-term effect of antibiotic use on the levels of resistance in the gut^47–49^, highlighting the need for more long-term resistome surveillance studies. For populations such as the one included in this study, this is particularly important.

Consistent with a pilot study in our setting^50^, the different storage methods used in this study did not significantly influence the microbiota. We also did not find any associations between diversity and sample consistency and weight. Further studies with larger sample sizes need to be conducted to explore the potential benefits of unrefrigerated sample transport for studies set in remote or resource-limited settings.

Exposure to indoor cooking fires rather than cigarette smoke was associated with a reduction in alpha diversity and significant bacterial community dissimilarity between samples, although no differentially abundant features could be identified. Wood and kerosene fires produce high levels of household pollutants, including a mixture of gases and particulate matter (PM). The WHO attributes 3.8 million deaths a year to household air pollution^51^, yet the relationship between PM and the microbiome is not well studied. Liu and colleagues recently reported that exposure to certain PM was associated with a reduction in alpha diversity^52^; however, results in animal studies have been conflicting^53,54^. Further studies are needed to improve our understanding of the impact of the use solid fuel and kerosene on gut microbiome related health in low- and middle-income countries.

In this study, deworming within 6 months of sample collection was associated with increased overall diversity and an increase in the abundance of *Haemophilus*. A Kenyan study determined that albendazole-based deworming resulted in an increase in Clostridiales and reduction of Enterobacterales; however the overall diversity decreased post-treatment^55^. In comparison, a study that investigated various anthelminthic agents did not observe any significant changes in alpha diversity but did find an association between combination tribendimidine and ivermectin therapy, and an increase in the phylum Bacteroidetes^56^. Similarly, alpha diversity was not affected by deworming therapy in hospitalised South African infants, but the bacterial gut microbiota communities of dewormed infants were found to be dissimilar to those who had not been dewormed^19^. Interestingly, a decrease in the relative abundance of Bacteroidetes has been associated with individuals who remained infected with helminths following deworming therapy^57^. As noted in this study, Yang et al. found that deworming resulted in a short-term increase in bacterial diversity, and additionally noted an increase in the probiotic *Bifidobacterium*, and decrease in *Fusobacteria*; however, this was dependent on the pre-treatment microbial profile^58^. They also suggested a possible link to increased secretory IgA (SIgA) following treatment^58^. In a recent study, people with SIgA deficiency were shown to have significantly less diverse microbiomes when compared to healthy controls^59^. SIgA is also known to aid in pathogen control and limit inflammation in the gut, thereby contributing to gut homeostasis^60^. However, the relationship between SIgA and deworming has not been established and needs further evaluation. There are no reports in the literature linking *Haemophilus* abundance to deworming. A study performed on South African infants determined that *Haemophilus* was a candidate gut pathogen associated with respiratory disease in infants^19^, but the clinical significance of *Haemophilus* outside of specific disease contexts is unknown. Further, we were only able to include 59 of 115 children in this analysis due to discrepant answers on the questionnaires, and did not evaluate the helminth infection status, which has been associated with specific microbiome profiles^61^. The numerous benefits of deworming in child health, such as control of anaemia and a lower risk of stunting and malnutrition, must also be considered and may mediate perceived negative microbiome effects. Like deworming, living with pets was also linked to an increase in gut microbial diversity. This relationship has previously been shown in infants, and changes in the abundance of a number of taxa have been associated with exposure to pets^62,63^; however, this was not seen in this study.

This cohort presented the rare opportunity to describe the microbiota of relatively healthy children from South African communities. The differences in the microbiota during the first 5 years of life in this setting are similar to what has been described elsewhere, and the abundance of taxa previously associated with good health indicates a low level of dysbiosis in this population. Those exposed to antibiotics and indoor wood/kerosene cooking fires were at the greatest risk for dysbiosis with significant losses in community diversity. This highlights the importance of continued and increased microbial surveillance in populations exposed to antibiotics and indoor cooking fires. Considering that it can be difficult to draw comparisons between 16S rRNA gene sequencing studies, due to the variance introduced by DNA extraction, sequencing and taxonomic profiling strategies, further functional studies are necessary to elucidate the full impact of environmental factors on the metabolic and immunological pathways connected to the microbiome. The extensive baseline metadata analysis performed here provides the ideal platform for future studies in this setting that will aim to identify the long-term impact of fluoroquinolones on the child gut microbiome and resistome; after the parent trial has been completed and the researchers can be unblinded.

## Supplementary Information

Supplementary Information.

## Data Availability

The sequencing data has been made available through the NCBI Sequence Read Archive under BioProject PRJNA692358.
