# Supplementary material for: Association between clinical and environmental factors and the gut microbiota profiles in young South African children
Source: Sci Rep. 2021 Aug 5;11:15895. doi: 10.1038/s41598-021-95409-5 (PMC8342602; doi:10.1038/s41598-021-95409-5)
Supplement: Supplementary file 1 — Supplementary Information. [file 41598_2021_95409_MOESM1_ESM.docx]

# Supplementary Material for:

# Association between clinical and environmental factors and the gut microbiota profiles in young South African children

K Nel Van Zyl^1^, AC Whitelaw^1,2,3^, AC Hesseling^4^, JA Seddon^4,5^, A-M Demers^4^, and M Newton-Foot^1,2^

1. Division of Medical Microbiology, Department of Pathology, Stellenbosch University, South Africa
2. National Health Laboratory Service, Tygerberg Hospital, Cape Town, South Africa
3. African Microbiome Institute, Stellenbosch University, South Africa
4. Desmond Tutu TB Centre, Department of Paediatrics and Child Health, Stellenbosch University, South Africa
5. Department of Infectious Diseases, Imperial College London, United Kingdom

# Methods

## DNA quality requirements

For sequencing at the Centre for Proteomic and Genomic Research (CPGR), DNA samples were required to have a concentration of at least 10 ng/μL and high purity (A_260/280_, 1.8 – 2.0; A_260/230_, 1.5 – 2.2) and integrity (≥10 Kb). Samples that failed to meet these standards after repeat extraction were subjected to ethanol-based purification, which was adapted from the QIAamp PowerFecal DNA Kit Handbook (Qiagen, Germany). 1/10 volume of 5 M NaCl was added to the final DNA eluate and inverted 3–5 times to mix. 2.5 volumes of ice cold 100% ethanol (kept at -20°C before use) was then added and inverted 3–5 times to mix, after which the tubes were incubate at room temperature for 5 minutes. The tubes were then centrifuged at 10,000 x *g* for 5 minutes at room temperature. The liquid was decanted, and any residual ethanol was removed with a pipette and allowed to air dry. The DNA was resuspended in 50 μL of the QIAamp kit elution buffer (C6).

The following samples (n=18) were subjected to purification following extraction: 3, 10, 11, 12, 13, 17, 18, 23, 37, 43, 45, 58, 70, 86, 89, 99, 102 and 105. One additional sample failed to meet the requirements after purification and was excluded from analysis.

## Sequencing controls

We included 7 sequencing controls in the Illumina sequencing run. The first was the ZymoBIOMICS Microbial Community DNA standard (Zymo Research, USA). Further, four extraction negative controls and two purification buffer controls were included to identify any possible introductions of DNA contamination:

- ZymoBIOMICS Microbial Community DNA standard
- A. Pooled Negatives Kit Lot #1, unspiked
- B. Pooled Negatives Kit Lot #1, spiked
- C. Pooled Negatives Kit Lot #2 (repeats), unspiked
- D. Pooled Negatives Kit Lot #2 (repeats), spiked
- E. NaCl buffer, unspiked
- F. NaCl buffer, spiked

The spike DNA was extracted from *Streptococcus* *pneumoniae* ATCC 49614. Spiking was performed to the mean concentration of the extracted sample DNA (~90 to 100 ng/μL) to simulate true contamination levels in the negative controls and buffer. Genomic DNA was extracted from the organism cultured in Brain Heart Infusion (BHI) broth (Sigma, Germany) overnight, using the QIAamp PowerFecal DNA Isolation Kit (Qiagen, Germany) as performed on the stool samples.

# Results

**Supplementary Table S1. Breastfeeding habits and day-care exposure of participants.**

| Category | **Group A** 0 to 1 years  n = 24 | **Group B** >1 to 2 years  n =25 | **Group C** >2 to 5 years  n =66 |
| --- | --- | --- | --- |
| Breastfed in first 6 months of life Yes No Unknown | 15 (62.5%)  8 (33.3%)  1 (4.2%) | 18 (72%)  7 (28%)  - | 45 (68.2%)  19 (28.8%)  2 (3%) |
| Exclusively breastfed Yes No Unknown | 13 (54.2%) 10 (41.6%)  1 (4.2%) | 16 (64%) 9 (36%) - | 36 (54.55%) 27 (40.91%) 3 (4.54%) |
| Duration of breastfeeding (months) Total Exclusively | 5 (3 – 6) 4.5 (3.5 – 6) | 6 (6 – 12) 6 (5.5 – 6) | 6 (3 – 12) 6 (5 – 8) |
| Age of solid food introduction (months) | 5 (4 – 6) | 6 (6 – 7) | 6 (4 – 7) |
| Day-care attendance | 2 (8.3%) | 5 (20%) | 30 (45.5%) |
| Hours in day-care | ND | ND | Median 8 hours  (min 4h – max 12h) |
| Number of children in day-care facility | ND | ND | Median 30 children (min 5 – max 250) |

Data expressed as number (%) or median (interquartile range), unless indicated otherwise.

ND = not determined due to limited samples in group

**Supplementary Table S2. Differences in alpha diversity between groups based on Shannon’s H and Faith’s PD metrics.**

| Category | **Shannon’s H** | | | **Faith’s PD** | | |
| --- | --- | --- | --- | --- | --- | --- |
|  | **Kruskal-Wallis test statistic** | **p-value** | **Corrected  p-value** | **Kruskal-Wallis test statistic** | **p-value** | **Corrected  p-value** |
| Demographics | | | | | | |
| Sex (Male vs Female) | 0.12 | 0.73 | - | 0.008 | 0.92 | - |
| Maternal HIV status (Positive vs Negative) | 0.18 | 0.67 | - | 0.26 | 0.61 | - |
| Age groups (years) 0 to 1 vs >1 to 2 0 to 1 vs >2 to 3 0 to 1 vs >3 to 4 0 to 1 vs >4 to 5 >1 to 2 vs >2 to 3 >1 to 2 vs >3 to 4 >1 to 2 vs >4 to 5 >2 to 3 vs >3 to 4 >2 to 3 vs >4 to 5 >3 to 4 vs >4 to 5 | 11.77  21.66  16.37  27.29  9.50  3.37  16.40  0.96  1.45  3.23 | 0.001  <0.001  <0.001  <0.001  0.002  0.066  <0.001  0.328  0.228  0.072 | 0.001  <0.001  <0.001  <0.001  0.003  0.09  <0.001  0.328  0.253  0.09 | 5.43  22.39  11.92  24.33  18.11  7.08  20.70  0.34  1.66  2.46 | 0.020  <0.001  0.001  <0.001  <0.001  0.008  <0.001  0.557  0.198  0.116 | 0.028  <0.001  0.001  <0.001  <0.001  0.013  <0.001  0.557  0.220  0.146 |
| Method of birth (NVD vs C/S) Group A Group B Group C | 0.50 0.02 0.19 | 0.48 0.88 0.66 | - - - | 0.24 2.4 0.03 | 0.62 0.12 0.87 | - - - |
| Premature birth (<37 weeks gestation) Group A Group B Group C | 0.20 0.39 0.06 | 0.65 0.53 0.81 | - - - | 0.20 0.17 0.32 | 0.65 0.68 0.57 | - - - |
| Breastfeeding (first six months of life) Group A Group B Group C | 0.05 0.36 0.28 | 0.82 0.55 0.60 | - - - | 0.94 0.03 1.23 | 0.33 0.87 0.27 | - - - |
| Exclusive breastfeeding Group A Group B Group C | 0.61 0.02 0.03 | 0.43 0.88 0.87 | - - - | 1.64 0.47 0.22 | 0.20 0.49 0.64 | - - - |
| Day-care exposure Group A Group B Group C | 0.70 0.15 0.12 | 0.40 0.70 0.72 | - - - | 1.44 0.21 1.57 | 0.23 0.64 0.21 | - - - |
| Clinical factors | | | | | | |
| Antibiotic receipt (<2 weeks from baseline) | 7.73 | 0.005 | - | 2.62 | 0.11 | - |
| Antibiotic receipt (<6 months from baseline) | 1.18 | 0.28 | - | 0.64 | 0.42 | - |
| Hospital admission (<6 months from baseline) | 2.59 | 0.11 | - | 3.09 | 0.08 | - |
| Hospital admission (first 6 months of life) | <0.001 | 0.98 | - | 0.75 | 0.39 | - |
| Visit to traditional healer (<6 months from baseline) | 6.47 | 0.01 | - | 7.89 | 0.005 | - |
| Dewormed (<6 months from baseline) | 4.79 | 0.029 | - | 3.42 | 0.06 | - |
| Vitamin A supplementation (<6 months from baseline, children >6 months) | 1.34 | 0.25 | - | 1.93 | 0.16 | - |
| Environmental factors | | | | | | |
| Cigarette smoke exposure from mother and/or household | 1.32 | 0.25 | - | 2.83 | 0.09 | - |
| Indoor cooking fire exposure | 8.17 | 0.004 | - | 6.13 | 0.01 | - |
| Pets (cats/dogs) in household | 4.49 | 0.03 | - | 4.21 | 0.04 | - |
| Sample storage  Fridge vs On ice Fridge vs Room temp On ice vs Room temp | 0.62 0.33 0.96 | 0.43 0.56 0.33 | 0.56 0.56 0.56 | 0.86 0.58 1.42 | 0.35 0.44 0.23 | 0.44 0.44 0.44 |
| Sample consistency  Liquid vs Semi-solid Liquid vs Solid Liquid vs Sticky Semi-solid vs Solid Semi-solid vs Sticky Solid vs Sticky | 1.99 1.29 1.55 0.07 1.51 0.99 | 0.16 0.26 0.21 0.79 0.22 0.32 | 0.38 0.38 0.38 0.79 0.38 0.38 | 2.60 1.64 1.21 0.005 0.57 0.35 | 0.11 0.20 0.27 0.95 0.45 0.55 | 0.54 0.54 0.54 0.95 0.67 0.67 |
|  |  |  |  |  |  |  |
| Socio-economic factors | | | | | | |
| Household structure Brick structure vs Tin shack Brick structure vs Shed Brick structure vs Prefab house Brick structure vs Backyard shack Tin shack vs Shed Tin shack vs Prefab house Tin shack vs Backyard shack Shed vs Prefab house Shed vs Backyard shack Prefab house vs Backyard shack | 1.12  0.03  0.03  1.77  0.20  0.11  0.70  0.07  1.00  0.20 | 0.29  0.87  0.87  0.18  0.65  0.74  0.40  0.80  0.32  0.65 | 0.87  0.87  0.87  0.87  0.87  0.87  0.87  0.87  0.87  0.87 | 0.50  0.84  0.00  1.64  0.20  0.07  1.16  0.07  1.00  0.20 | 0.48  0.36  0.96  0.20  0.65  0.79  0.28  0.80  0.32  0.65 | 0.88  0.88  0.96  0.88  0.88  0.88  0.88  0.88  0.88  0.88 |
| Ablution type  Bucket system vs Flush toilet in the house  Bucket system vs Exclusive flush toilet outside  Bucket system vs Pit latrine  Bucket system vs Shared flush toilet  Bucket system vs VIP latrine  Flush toilet in the house vs Exclusive flush toilet outside  Flush toilet in the house vs Pit latrine  Flush toilet in the house vs Shared flush toilet  Flush toilet in the house vs VIP latrine  Exclusive flush toilet outside vs Pit latrine  Exclusive flush toilet outside vs Shared flush toilet  Exclusive flush toilet outside vs VIP latrine  Pit latrine vs Shared flush toilet  Pit latrine vs VIP latrine  Shared flush toilet vs VIP latrine | 0.13  0.27  4.15  0.00  1.85  0.03  4.59  0.13  4.37  2.89  0.45  4.41  1.29  0.33  2.58 | 0.72  0.61  0.04  0.95  0.17  0.85  0.03  0.71  0.04  0.09  0.50  0.04  0.26  0.56  0.11 | 0.83  0.83  0.16  0.95  0.37  0.92  0.16  0.83  0.16  0.27  0.83  0.16  0.48  0.83  0.27 | 0.41  0.15  0.46  0.09  1.85  0.08  1.89  0.10  5.18  0.49  0.16  3.24  1.29  0.08  2.29 | 0.52  0.70  0.50  0.76  0.17  0.78  0.17  0.75  0.02  0.48  0.69  0.07  0.26  0.77  0.13 | 0.78  0.78  0.78  0.78  0.52  0.78  0.52  0.78  0.34  0.78  0.78  0.52  0.64  0.78  0.52 |
| Drinking water supply  Piped water from a public shared tap vs in residence Piped water from a public shared tap vs exclusive tap outside Piped water in the residence vs exclusive tap outside | 0.13  0.99  2.42 | 0.72  0.32  0.12 | 0.72  0.48  0.36 | 0.18  1.08  1.25 | 0.67  0.30  0.26 | 0.67  0.45  0.45 |

* For factors with >2 groups, the corrected p-values have been given (Benjamini-Hochberg False Discovery Rate (BH-FDR) multiple test correction)

Five samples were excluded following rarefaction and samples with no data for a particular factor were excluded from that analysis.

**Supplementary Table S3. Spearman correlation for the association between Shannon’s H and numerical data.**

| Category | **Spearman test statistic** | **p-value** |
| --- | --- | --- |
| Stool weight (g) | 0.09 | 0.35 |
| Duration of breastfeeding (months) Group A Group B Group C | 0.20 0.15 0.02 | 0.52 0.59 0.90 |
| Duration of exclusive breastfeeding (months) Group A Group B Group C | -0.15 0.17 -0.05 | 0.66 0.57 0.78 |
| Age solid food was introduced (months) Group A Group B Group C | -0.23 -0.33 0.16 | 0.37 0.15 0.23 |
| Group C: Age day-care was started (months) | 0.09 | 0.63 |
| Group C: Size of day-care group | -0.15 | 0.42 |
| Group C: Hours a day spent in day-care | 0.14 | 0.46 |

Five samples were excluded following rarefaction and samples with no data for a particular factor were excluded from that analysis.


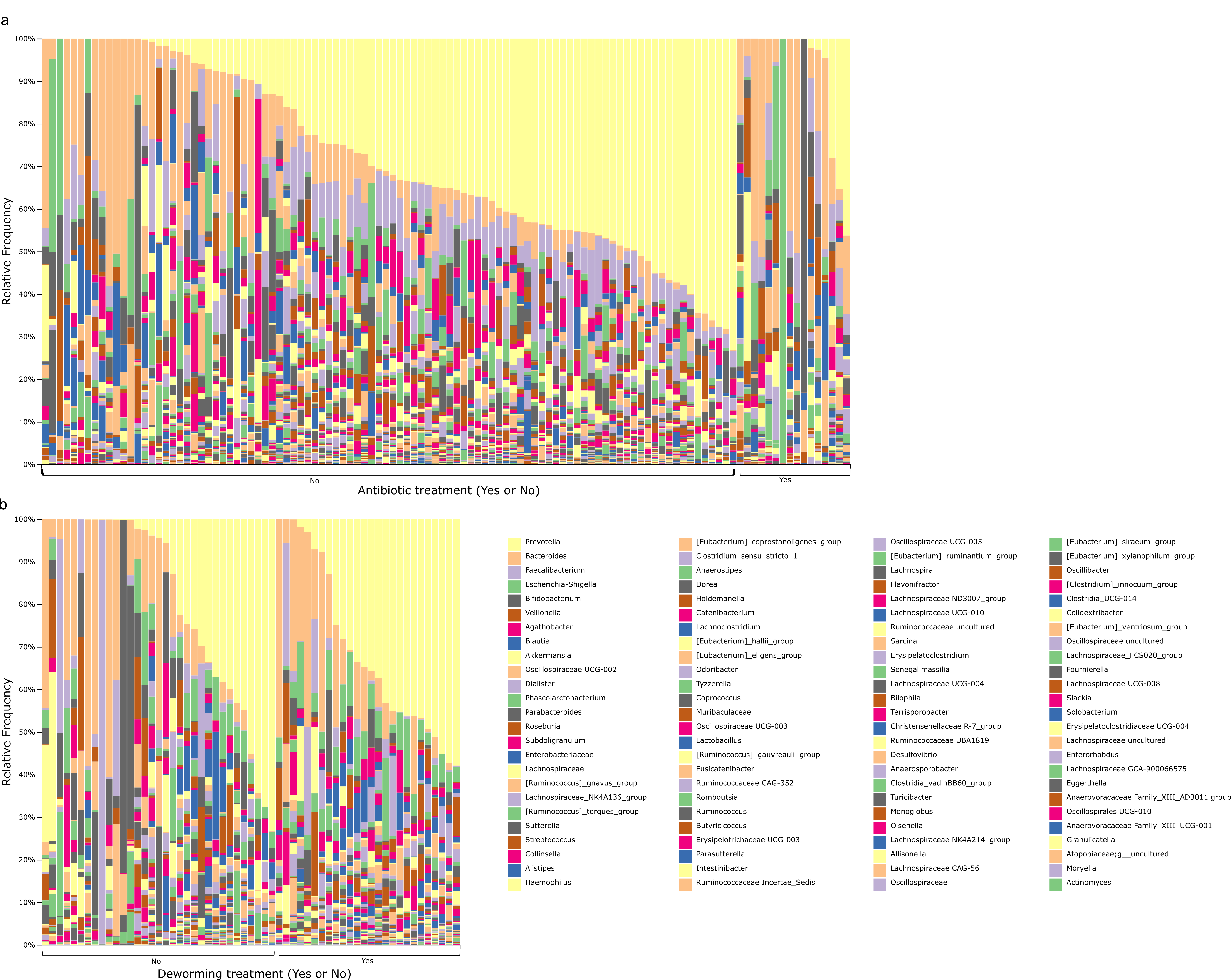


**Supplementary Figure S1. Differences in the composition of gut bacteria between children who received antibiotics, and children who received deworming therapy before baseline.** a. Antibiotic therapy within two weeks of baseline. b. Deworming therapy within 6 months of baseline. The legend shows the identified taxa in order of abundance from top to bottom, starting from the leftmost column. For example, *Prevotella*, *Bacteroides* and *Faecalibacterium* were the top 3 most abundant genera.
